# Supplementary material for: Fourth Dose of mRNA COVID-19 Vaccine Transiently Reactivates Spike-Specific Immunological Memory in People Living with HIV (PLWH)
Source: Biomedicines. 2022 Dec 15;10(12):3261. doi: 10.3390/biomedicines10123261 (PMC9775459; doi:10.3390/biomedicines10123261)
Supplement: Supplementary file 1 [file biomedicines-10-03261-s001.zip › biomedicines-1980850-supplementary.pdf]

Supplementary Appendix

Table of Contents

Supplemental Table 1. List of all fluorochrome-conjugated antibodies used for flow cytometric analysis of immunophenotyping of lymphoid cell subsets.

Supplemental Table 2. List of all fluorochrome-conjugated antibodies used for flow cytometric analysis of antigen-specific and polyclonal T cells.

Supplemental Table 3. List of all fluorochrome-conjugated antibodies used for flow cytometric analysis of antigen-specific B cells.

Supplemental Table 4. List of all fluorochrome-conjugated antibodies used for flow cytometric analysis of antigen-specific B cells.

Supplemental Figure 1. Gating strategy for immunophenotyping of lymphoid cell subsets.

Supplemental Figure 2. Gating strategy for the identification of SARS-CoV-2-specific T cells.

Supplemental Figure 3. Gating strategy for the identification of Spike-specific B cells.

Supplemental Table 1. List of all fluorochrome-conjugated antibodies used for flow cytometric analysis of immunophenotyping of lymphoid cell subsets.

Antigen	Fluorochrome	Clone	Company
HLA-DR	FITC	L243	BDBioscience
CD56	PE	NCAM16.2	BDBioscience
CD16	PerCP 5.5	3G8	BDBioscience
CD4	PE-Cy7	SK3	BDBioscience
CD19	APC	SJ25C1	BDBioscience
CD8	APC-H7	SK1	BDBioscience
CD3	V450	ICHT1	BDBioscience
CD45	V500	2D1	BDBioscience

Supplemental Table 2. List of all fluorochrome-conjugated antibodies used for flow cytometric analysis of antigen-specific and polyclonal T cells.

Antigen	Fluorochrome	Clone	Company
TNF-α	FITC	6401.1111 B	BDBioscience
CD154	PE	TRAP1	BDBioscience
CD3	PerCP	SK7	BDBioscience
CD4	PECy-7	SK3	Invitrogen
CD8	SB600	SK1	eBioscience™
IL-2	APC	MQ1-17H12	BDBioscience
IFN-γ	Pacific Blue	B27	BioLegend
L/D	Fixable Viability Stain 780		BDBioscience

Supplemental Table 3. List of all fluorochrome-conjugated antibodies used for flow cytometric analysis of antigen-specific B cells.

Antigen	Fluorochrome	Clone	Company
CD19	APC-Vio770	LT19	Miltenyi
CD27	VioBright FITC	M-T271	Miltenyi
IgA	VioGreen	IS11-8E10	Miltenyi
IgG	VioBlue	IS11-3B2.2.3	Miltenyi
CD14	PerCP	TÜK4	Miltenyi
IgM	APC	PJ2-22H3	Miltenyi
CD3	PerCP	BW264/56	Miltenyi
7AAD			Miltenyi
Spike	PE		Miltenyi
Spike	PE-Vio770		Miltenyi

Supplemental Table 4. List of all fluorochrome-conjugated antibodies used for flow cytometric analysis of antigen-specific B cells.

**Antigen**	**Fluorochrome**	**Clone**	**Company**
CD19	APC-Vio770	LT19	Miltenyi
CD27	VioBright FITC	M-T271	Miltenyi
Wuhan WT Spike	APC		Miltenyi
Wuhan WT Spike	VioBlue		Miltenyi
CD14	PerCP	TÜK4	Miltenyi
CD3	PerCP	BW264/56	Miltenyi
7AAD			Miltenyi
Spike Omicron B1.1.529	PE		Miltenyi
Spike Omicron B1.1.529	PE-Vio770		Miltenyi

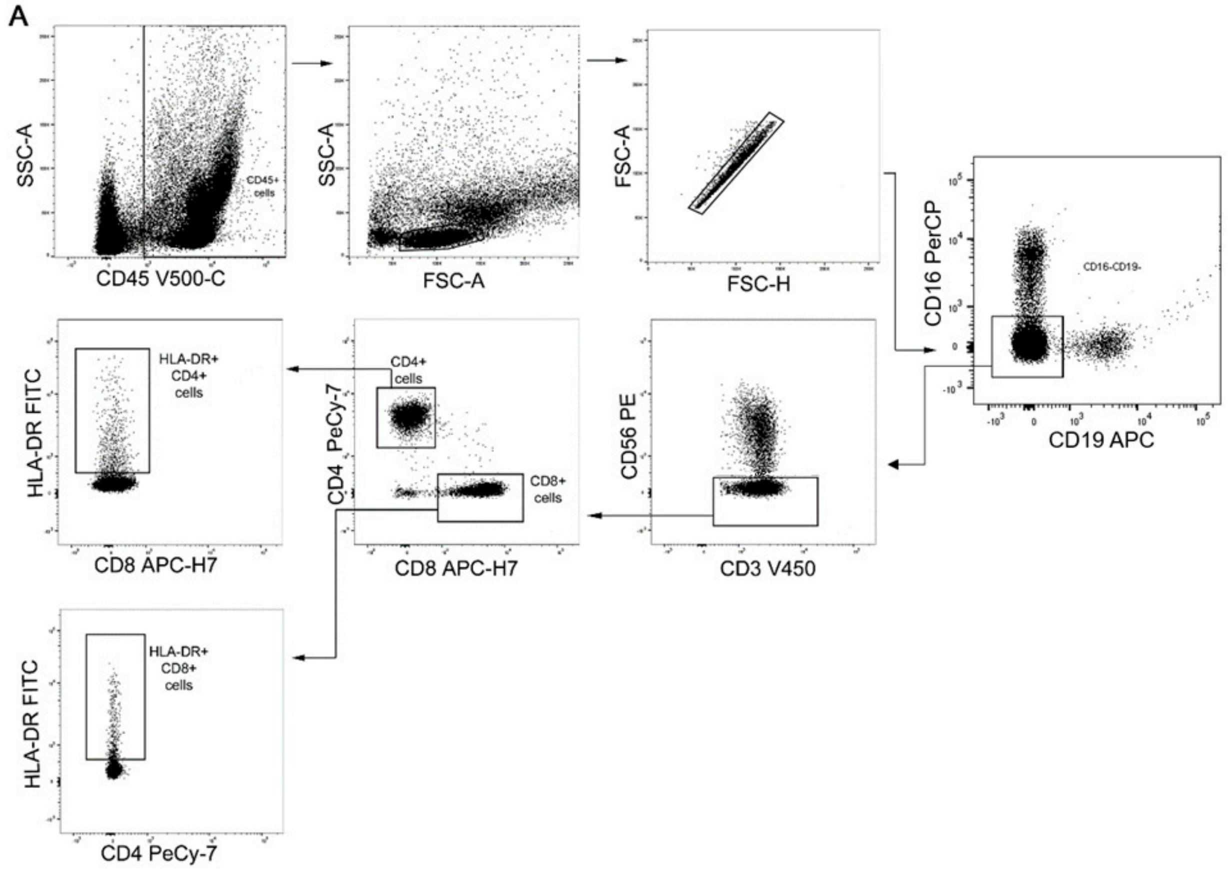

Supplemental Figure 1. Gating strategy for immunophenotyping of lymphoid cell subsets.

(**A**) PBMCs were gated based on physical parameter SSC and CD45 surface expression, lymphocytes were then gated basing on physical parameters (SSC, FSC). Doublets were removed using FSC-A and FSC-A parameters. CD16-CD19- and CD56-CD3+ cells were identified. In the resulting CD3+ population, CD4+ and CD8+ T lymphocytes were identifiedAmong these, we identified HLA-DR expressing cells.

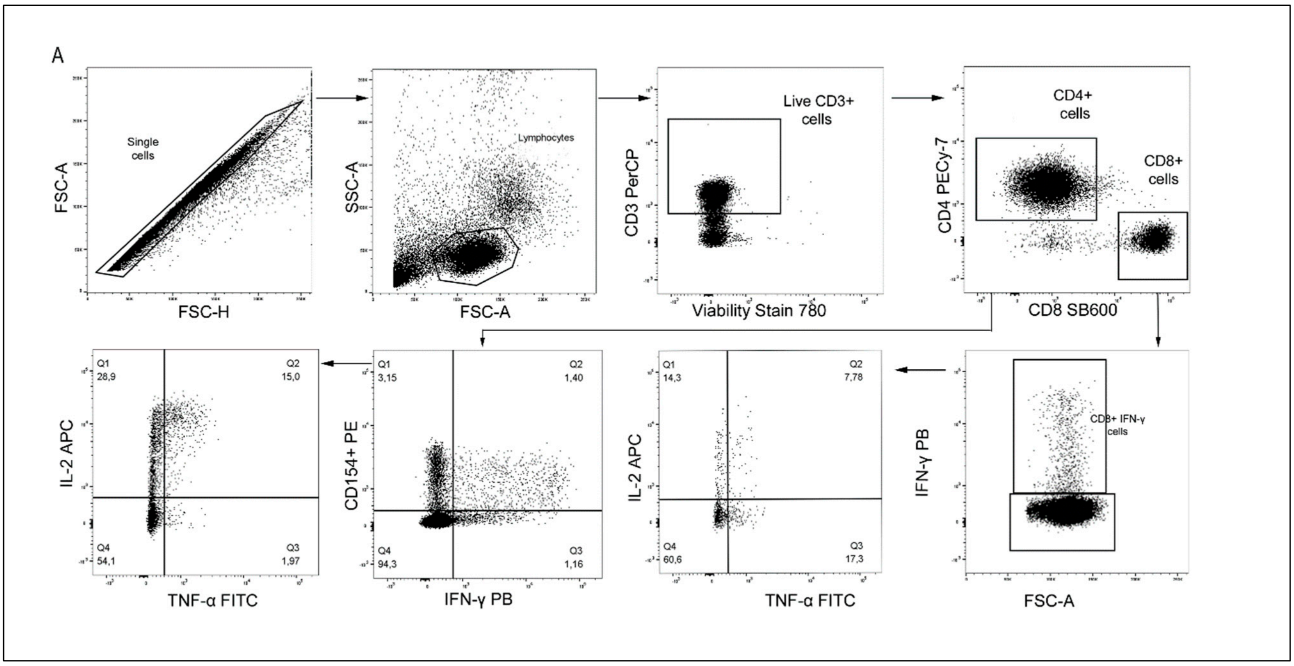

Supplemental Figure 2. Gating strategy for the identification of SARS-CoV-2-specific T cells.

(A) Doublets were removed using FSC-A and FSC-A parameters and lymphocytes were gated based on physical parameters (FSC-SSC). Dead cells were excluded using viability stain 780. T cells were identified as CD3+. We then identified CD4+ and CD8+ T cells. CD4+ T cells were evaluated for CD154, IFN-γ, TNF-α and IL-2 expression. CD8+ T cells were evaluated for IFN-γ, TNF-α and IL-2 expression.

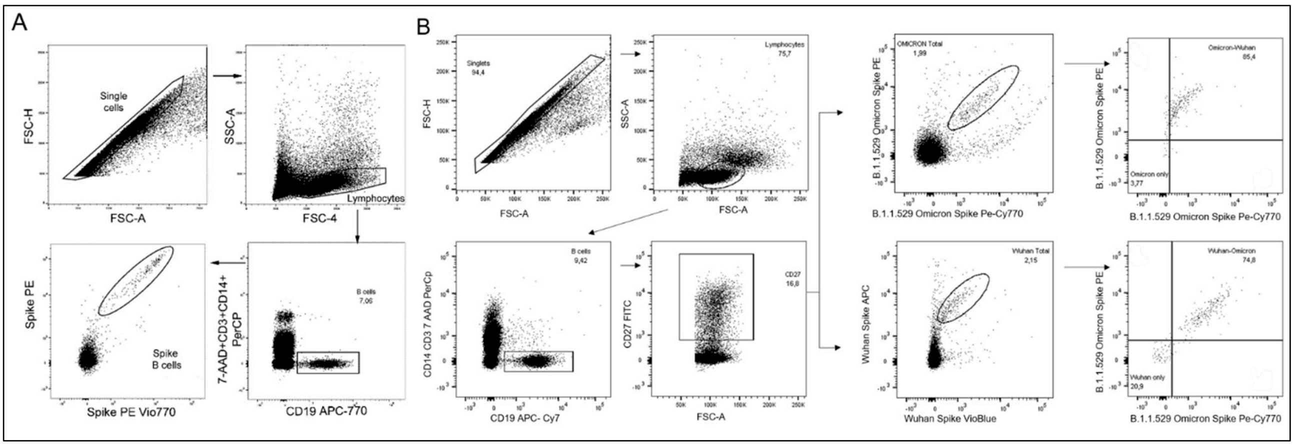

Supplemental Figure 3. Gating strategy for the identification of Spike-specific B cells.

(**A**) Doublets were removed using FSC-A and FSC-A parameters and lymphocytes were gated based on physical parameters (FSC-SSC). PerCP was used as dump channel for the exclusion of dead cells (7AAD), CD3+ T cells and CD14 monocytes. B cells were identified as CD19+. B cells binding PE- and PE Vio770-conjugated spike protein were then identified as Wuhan (WT) Spike-specific. (**B**) Doublets were removed using FSC-A and FSC-A parameters and lymphocytes were gated based on physical parameters (FSC-SSC). PerCP was used as dump channel for the exclusion of dead cells (7AAD), CD3+ T cells and CD14 monocytes. Memory B cells were identified as CD19+CD27+. B cells binding PE- and PE Vio770-conjugated spike protein were then identified as B.1.1.529 (Omicron) Spike-specific and B cells binding APC- and Pacific Blue-conjugated Spike protein were then identified as Wuhan (WT) Spike-specific.
